# Congenital imprinting disorders: EUCID.net - a network to decipher their aetiology and to improve the diagnostic and clinical care

**DOI:** 10.1186/s13148-015-0050-z

**Published:** 2015-03-14

**Authors:** Thomas Eggermann, Irène Netchine, I Karen Temple, Zeynep Tümer, David Monk, Deborah Mackay, Karin Grønskov, Andrea Riccio, Agnès Linglart, Eamonn R Maher

**Affiliations:** Department of Human Genetics, RWTH Aachen, Aachen, 52074 Germany; INSERM, UMR_S 938, CDR Saint-Antoine, Paris, F-75012 France; UMR_S 938, CDR Saint-Antoine, UPMC Univ Paris 06, Sorbonne Universites, Paris, F-75012 France; Pediatric Endocrinology, 3APHP, Armand Trousseau Hospital, Paris, 75012 France; Human Genetics and Genomic Medicine, Faculty of Medicine University of Southampton, Wessex Clinical Genetics Service, Princess Anne Hospital, Coxford Road, Southampton, SO16 5YA UK; Clinical Genetic Clinic, Kennedy Center, Rigshospitalet, Copenhagen University Hospital, Glostrup, 2600 Denmark; Imprinting and Cancer Group, Cancer Epigenetic and Biology Program (PEBC), Institut d’Investigació Biomedica de Bellvitge (IDIBELL), Hospital Duran i Reynals, 08907 Barcelona, Spain; DiSTABiF, Seconda Università degli Studi di Napoli, 81100 Caserta, Italy; Institute of Genetics and Biophysics—ABT, CNR, Napoli, Italy; Endocrinology and Diabetology for Children and Reference Center for Rare Disorders of Calcium and Phosphorus Metabolism, Bicêtre Paris Sud, APHP, Le Kremlin-Bicêtre, 94276 Paris France; INSERM U986, INSERM, Le Kremlin-Bicêtre, 94276 Paris, France; Department of Medical Genetics, NIHR Cambridge Biomedical Research Centre, University of Cambridge, Cambridge, CB2 OXY UK; Department of Human Genetics, University Hospital, RWTH Aachen, Pauwelsstr. 30, 52074 Aachen, Germany

**Keywords:** Imprinting disorders, Imprinted genes, Epimutation, Uniparental disomy, EUCID.net, Networking

## Abstract

Imprinting disorders (IDs) are a group of eight rare but probably underdiagnosed congenital diseases affecting growth, development and metabolism. They are caused by similar molecular changes affecting regulation, dosage or the genomic sequence of imprinted genes. Each ID is characterised by specific clinical features, and, as each appeared to be associated with specific imprinting defects, they have been widely regarded as separate entities. However, they share clinical characteristics and can show overlapping molecular alterations. Nevertheless, IDs are usually studied separately despite their common underlying (epi)genetic aetiologies, and their basic pathogenesis and long-term clinical consequences remain largely unknown. Efforts to elucidate the aetiology of IDs are currently fragmented across Europe, and standardisation of diagnostic and clinical management is lacking. The new consortium EUCID.net (European network of congenital imprinting disorders) now aims to promote better clinical care and scientific investigation of imprinting disorders by establishing a concerted multidisciplinary alliance of clinicians, researchers, patients and families. By encompassing all IDs and establishing a wide ranging and collaborative network, EUCID.net brings together a wide variety of expertise and interests to engender new collaborations and initiatives.

## Review

### Introduction

**Figure 1:**
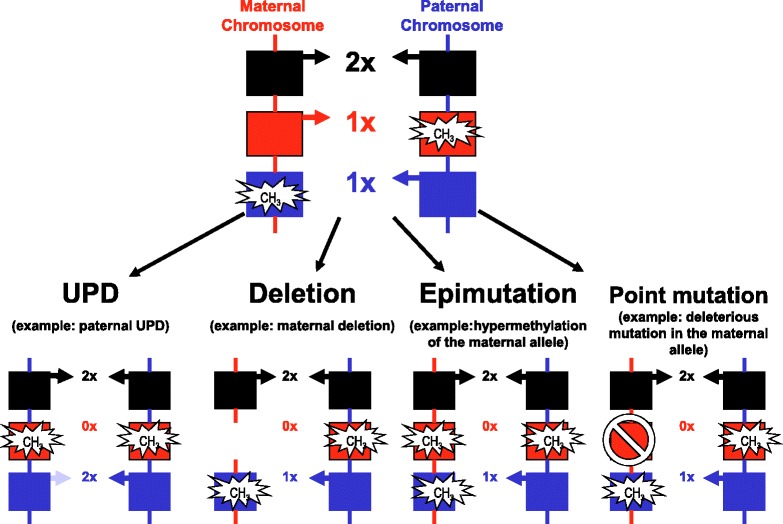
**The four molecular mechanisms of IDs, resulting in a disturbed expression of imprinted genes.**

Imprinting disorders (IDs) are a group of eight rare congenital diseases affecting growth, development and metabolism with a lifelong impact on patients’ quality of life. They are caused by changes in gene regulation (‘epigenetic mutation’), gene dosage and - rarely - in gene or genomic sequences (‘genetic mutation’) (Figure 1). The term genomic imprinting describes the expression of specific genes in a parent-of-origin-specific manner - that is, they are expressed only from the maternal or from the paternal gene copy, but not biparentally (for review: [1]). The underlying epigenetic basis does not involve the DNA sequence itself, but regulatory mechanisms that ensure the transmission of specific gene expression patterns from one cell generation to another, ensuring the maintenance of cellular identity.

So far, more than 60 human genes have been shown to be imprinted, but there are probably many more (for review: [2]). The normal imprinting marks are inherited from the parental gametes and are then maintained in the somatic cells of an individual. Their programming is subject to an imprinting cycle during life which leads to a reprogramming at each generation (for review: [3]): In early development, methylation of the mammalian genome is comprehensively remodelled, but imprinting marks are exempt from developmental reprogramming; instead, they are erased in the germ line and re-established according to the sex of the contributing parent for the next generation. Many genes regulated by genomic imprinting are found in clusters, that is, imprinted loci often comprise multiple genes under coordinated control. At the molecular level, the expression of genes within imprinted regions is influenced by specific patterns of DNA methylation, by changes in chromatin structure and by post-translational histone modifications, collectively designated as epigenetic regulation (for review: [4,5]).

The epigenetic machinery is extremely complex and results in a unique transcriptional activity of different cells with identical DNA sequences. Indeed, this carefully orchestrated interplay is prone to various disturbances resulting in distinct pathological courses, for example, malignant tumours or - in the case of parentally imprinted genes - IDs. In IDs, the regulation of imprinted genes can be disturbed by four different molecular alterations: genomic imbalances (duplications/deletions), uniparental disomy (UPD; the inheritance of both homologos of a chromosomal pair from only one parent), epimutations (disturbed methylation) or point mutations in an imprinted gene. Whereas in the majority of ID patients, only the disease-specific loci are affected, an increasing number of ID patients are reported showing a disturbed methylation at multiple differentially methylated regions (DMRs), the so-called multilocus imprinting disturbances (MLID) (see below). The extreme examples of unbalanced imprinting patterns are genome-wide UPDs, that is, the whole genome is inherited only from the father or from the mother. In both cases, the resulting conception is not viable. However, mosaic genome-wide UPD has been reported to be compatible with life (for review: [6]).

### The known imprinting disorders

Most patients with one of the currently established IDs are diagnosed in early childhood. In contrast, the diagnosis in the prenatal workup or puberty or adulthood is often hampered because the clinical spectrum is broad, and some features are subtle, overlapping and transient. As a result, some IDs are probably mis- and underdiagnosed.

Each ID is characterised by specific clinical features, and as they appeared to be associated with specific imprinting defects, they have been regarded as separate entities. Indeed, the majority of IDs have some shared clinical characteristics (Table 1), that is:

**Table 1:** **Overview on the clinical and molecular characteristics of the currently known eight IDslocalization**

**Imprinting disorder**	**Alternative name/acronym**	**Frequency**	**OMIM**	**Chromosomes** ^**a**^ **/imprinted regions**	**Type of mutation/epimutation**	**MLID**	**Detection rate**	**Main clinical features**
Transient neonatal diabetes mellitus	TNDM	1/300,000	601410	6q24^a^: *ZAC1/HYMA1*	upd(6)pat		40%	IUGR, transient diabetes, hyperglycemia without ketoacidosis, macroglossia, omphalocele
					Paternal duplications		40%	
					Methylation defects	Approximately 50%	20%	
Silver-Russell syndrome	Russell-Silver syndrome, SRS, RSS	1/75,000-1/100,000	180860	7^a^	upd(7)mat	One case^b^	Approximately10%	IUGR/PNGR, rel. macrocephaly, hemihypotrophy, triangular face, feeding difficulties
				11p15^a^:	upd(11p15)mat		Single cases	
					Maternal duplication		<1%	
				*IGF2/H19*	Hypomethylation	7% to 10%	>38%	
				*CDKN1C*	Point mutations		One family reported	
Beckwith-Wiedemann syndrome	Wiedemann-Beckwith syndrome, EMG syndrome, BWS	1/15,000	130650	11p15^a^:	upd(11p15)pat		Approximately 20%	Prenatal and postnatal overgrowth, organomegaly, macroglossia, omphalocele, neonatal hypoglycemia, hemihypertrophy, increased tumour risk
					Chromosomal aberrations		2% to 4%	
				ICR1*: IGF2/H19;*	Hypermethylation		5% to 10%	
				ICR2*:KCNQ1*	Hypomethylation	Approximately 25%	40% to 50%	
				*CDKN1C*	Point mutations		5% (sporadic) 40% to 50% (families)	
Kagami-Ogata syndrome	KOS14, upd(14)pat syndrome	Not known	608149	14q32^a^: *DLK1/GTL2*	upd(14)pat	Not yet reported	?	IUGR, polyhydramnion, abdominal and thoracal wall defects, bell-shaped thorax, coat-hanger ribs
					Aberrant methylation			
Temple syndrome	TS14, upd(14)mat syndrome	Not known		14q32^a^: *DLK1/GTL2*	upd(14)mat	One case^b^	?	IUGR/PNGR, Hypotonia, feeding difficulties in infancy, truncal obesity, scoliosis, precocious puberty
					Paternal deletion			
					Aberrant methylation			
Prader-Willi syndrome	Prader-Labhart-Willi-syndrome, PWS	1/25,000	176270	15q11-q13^a^	Paternal deletion	One case with PWS and BWS features	70%	PNGR, mental retardation, neonatal hypotonia, hypogenitalism, hypopigmentation, obesity/hyperphagia
		-1/10,000			upd(15)mat		<30%	
					Aberrant methylation		Approximately 1%	
Angelman syndrome	Happy puppet syndrome, AS	1/20,000	105830	15q11-q13^a^:	Maternal deletion	Not yet reported	70%	Mental retardation, microcephaly, no speech, unmotivated laughing, ataxia, seizures, scoliosis
		-1/12,000			upd(15)pat		1% to 3%	
					Aberrant methylation		Approximately 4%	
				*UBE3A*	Point mutations		10% to 15%	
Pseudohypo-parathyroidism	PHP1B, PHP1C and PHP1A	Not known	603233	20q13^a^	Maternally inherited deletions		Not yet reported	Resistance to PTH and other hormones
			612462	*GNAS*	Causing aberrant methylation			Albright hereditary osteodystrophy
			103580		Isolated epimutations	12.5%		Subcutaneous ossifications
					upd(20)pat			Feeding behaviour anomalies
					Maternal and paternal heterozygous loss of function mutations in the coding sequence of *GNAS*			Abnormal growth patterns

As listed in the second column, for several IDs, different names have been proposed. To reach a consensus on a common nomenclature of IDs, EUCID.net has decided to use the disorders names listed on the left (see www.imprinting-disorders.eu). IUGR, intrauterine growth retardation; PNGR, postnatal growth retardation. ^a^Chromosomes. ^b^Case [27] carries both upd(7)mat and an TS14 epimutations.

- prenatal and/or postnatal growth retardation or prenatal and postnatal overgrowth;
- hypo- or hyperglycemia;
- abnormal feeding behaviour in early childhood and later; and
- behavioural difficulties in childhood.

#### Transient neonatal diabetes mellitus

Transient neonatal diabetes mellitus (TNDM) is a rare disease, characterised as its name implies by transient hyperglycaemia. In addition, IUGR, macroglossia and abdominal wall defects are common. Insulin therapy is required for an average of 3 months; afterwards, the diabetes resolves, but later in life, the majority of TNDM patients develop type 2 diabetes. TNDM is associated with an overexpression of *PLAGL1/ZAC* in 6q24, a maternally imprinted gene. It encodes a zinc-finger protein which binds DNA and hence influences the expression of other genes (for review: [7,8]).

#### Silver-Russell syndrome

Silver-Russell syndrome (SRS) is mainly characterised by prenatal and postnatal growth restriction with relative macrocephaly at birth, severe feeding difficulties during early childhood and typical facial gestalt and, in many cases, asymmetry. The genetic basis of SRS is heterogeneous. In approximately 10% of SRS patients, a maternal UPD for chromosome 7 [upd(7)mat] can be found (for review: [9,10]). More than 40% of SRS patients show a hypomethylation of the ICR1 DMR in the imprinted region 11p15. In single cases, genomic alterations in 11p15 chromosomal region have been reported (for example, maternal duplications). Additionally, numerous (submicroscopic) disturbances of other chromosomes than 7 and 11 have been described in SRS patients; thus, screening for cryptic genomic imbalances is indicated after exclusion of upd(7)mat and 11p15 epimutations [11,12]. Furthermore, there is an overlap with Temple syndrome (upd(14)mat). The genes causing the SRS phenotype on chromosomes 7 and 11 are currently unknown, but a role of *IGF2* and *CDKN1C* in 11p15 has been suggested.

#### Beckwith-Wiedemann syndrome

Beckwith-Wiedemann syndrome (BWS) was initially called EMG syndrome from its three main features of exomphalos, macroglossia and (neonatal) gigantism. In 5% to 7% of children, embryonal tumours (most commonly Wilms tumour) are diagnosed. The clinical diagnosis of BWS is often difficult due to its variable presentation and the phenotypic overlap with other overgrowth syndromes (for review: [13,14]). In nearly 70% of BWS patients, an altered expression or mutations of several loci in 11p15 can be observed (including the ICR1 and ICR2 DMRs). ICR2 hypomethylation account for about 50% of the cases, upd(11p15)pat is the second most frequent molecular aberration, while ICR1 hypermethylation is less frequent (2% to 7%). Most BWS cases are sporadic, but familial inheritance is observed in 15% of all cases. Microdeletions/duplications or point mutations at the ICRs are usually found in familial BWS with aberrant 11p15 methylation, while *CDKN1C* mutations are frequent in familial cases with normal 11p15 methylation [15,16]. These BWS pedigrees resemble that of an autosomal dominant inheritance but with incomplete penetrance depending on the sex of the inheriting parent. A genotype/epigenotype-phenotype correlation has recently been established for BWS [17]: hemihypertrophy is strongly associated with upd(11)pat, exomphalos with ICR2 hypomethylation and *CDKN1C* mutations, and, most importantly, the risk of Wilms tumour is significantly higher in ICR1 hypermethylation and upd(11)pat than in the other molecular subgroups. In BWS, the determination of the molecular subtype is therefore important for an individual prognosis and therapy. Nevertheless, the phenotypic transitions are fluid, and testing for all molecular subtypes should be offered in patients with BWS features.

#### Temple syndrome (TS14) [upd(14)mat]) and Kagami-Ogata syndrome (KOS14) [upd(14)pat]

TS14 (upd(14)mat and WGS (upd(14)pat) were described in 1991 by Temple *et al*. and Wang *et al*. [18,19], respectively. However, the frequencies of both syndromes are currently unknown. Both IDs were first detected in patients carrying balanced Robertsonian translocations. Considering the most important formation mechanism of UPD via trisomy rescue, this observation was consequent because Robertsonian translocations are prone to trisomic offspring. More recently, several cases have been described with microdeletions affecting 14q32 or with isolated methylation anomalies affecting the imprinting control region [20,21].

Among other clinical signs, TS14 is characterised by prenatal and postnatal growth retardation, muscular hypotonia, feeding difficulties in early childhood, truncal obesity and early onset of puberty. TS14 patients show clinical features overlapping with PWS and SRS, and thus, screening for chromosome 14q32 should be performed in patients with PWS- and SRS-like phenotypes after exclusion of the specific (epi)mutations.

KOS14 is associated with polyhydramnios, a characteristic small, bell-shaped thorax, abdominal wall defects and a severe developmental delay. Many patients have been reported to die *in utero* or in the first months of life, but exceptions exist.

For both syndromes, the role of an altered *RTL1* and *DLK1* expression has been suggested [21].

#### Angelman and Prader-Willi syndromes

Both Angelman syndrome (AS) and Prader-Willi syndrome (PWS) are caused by (epi)mutations in 15q11-q13. The lack of the paternal contribution of this region results in PWS, while lack of the maternal contribution leads to AS. Both AS and PWS patients are mentally retarded, but the remaining clinical signs are different. PWS is clinically characterised by neonatal hypotonia and failure to thrive in infancy, with subsequent development of hyperphagia and obesity (for review: [22]). Approximately 70% of individuals with PWS have an interstitial deletion of the paternal 15q11-q13 allele, 20% to 30% have maternal UPD of 15q11-q13 while <1% have an imprinting defect (either primary or secondary). As mentioned before, analysis for TS14 should be considered in PWS patients without chromosome 15 disturbances.

AS patients exhibit microcephaly, ataxia, seizures, absence of speech and sleep disorder (for review: [23]). Deletion of the maternal copy of 15q11-q13 is observed in approximately 70% of individuals with AS, paternal UPD in 7% to 10%, while 3% have an imprinting defect (either primary or secondary). Around 10% have mutations in *UBE3A*.

Due to the high percentage of microdeletions in 15q11-q13 in both syndromes, AS and PWS also belong to the so-called microdeletion syndromes, a group of congenital disorders caused by a chromosomal deletion spanning several genes but too small to be detected by conventional cytogenetic.

#### Pseudohypoparathyroidism

Pseudohypoparathyroidism (PHP) is a group of disorders united by parathyroid hormone (PTH) resistance in the kidney, that is, pseudohypoparathyroidism. Most cases of PHP belong to the type 1, that is, are caused by genetic or epigenetic alterations at the imprinted *GNAS* locus. PHP1A comprises patients affected with resistance to PTH and thyroid stimulating hormone (TSH) and features of obesity and Albright’s hereditary osteodystrophy including short stature, brachydactyly, ectopic ossifications and mental retardation. PHP1A is due to loss of function mutations in the maternal allele of the *GNAS* gene. Paternal GNAS mutations are associated with AHO, no hormonal resistance and no obesity. In contrast, the phenotype of most PHP1B patients is limited to renal PTH resistance and in some cases, mild TSH resistance. Few patients with PHP1B display some features of Albright’s hereditary osteodystrophy. Patients with PHP1B share a loss of methylation at the A/B DMR of *GNAS*, likely leading to the downregulated expression of the *GNAS*-Gsa transcript in imprinted tissues. Some patients carry additional epigenomic changes along the *GNAS* locus. About 20% of PHP1B are inherited and due to deletions of *GNAS* imprinting control regions. The remaining 80% are sporadic. A small subset is due to paternal UPD of chromosome 20q, yet the vast majority are still of unknown cause. While obesity and short stature are long known features of PHP1A, it became only recently apparent that growth and metabolism are affected in both paternal and maternal epi/genetic alterations of the GNAS locus (for review: [24,25]).

### Molecular alterations in IDs

In nearly all known IDs, the same classes of molecular changes are detectable. Broadly, the several mechanisms have an underlying genetic lesion, but a considerable number have no identifiable genetic cause, and are reproductive, stochastic or epigenetic in origin (Figure 1).

The genetic lesions include:

- chromosomal deletions, duplications and rearrangements;
- intragenic mutations in imprinted genes.

In familial cases of these genetically caused IDs, parent of origin dependence of expression results in apparent non-Mendelian inheritance.

The non-genetic causes consist of:

c) UPD (that is, the inheritance of both chromosomal homologos from the same parent);
d) epimutations (that is, aberrant methylation without alteration of the genomic DNA sequence).

It is noteworthy that non-genetic aberrations may occur post-zygotically, resulting in a mosaic distribution. Mosaicism can obscure genotype-phenotype correlation, and is also associated with somatic asymmetry, and discordant monozygotic twinning.

For genetic counselling of ID families, the knowledge of the nature of the mutation or epimutation subtype is essential to delineate exact risk figures. Whereas the recurrence risk is generally low in the case of epimutations and UPD, patients/carriers with submicroscopic deletions or duplications might have a 50% risk of conceiving a child with an ID, depending on the sex of the contributing patient. However, in each case, genetic professionals are advised to continually update their knowledge for each disease.

Each of the currently known IDs was initially reported to be associated with molecular alterations at specific chromosomal loci. They have therefore been regarded as separate entities, but - as mentioned before - with the growing data on IDs, it becomes apparent that they share both genetic properties and clinical features. This can cause uncertainty determining which molecular tests to perform and with what priority, particularly for patients with growth restriction. Moreover, the clinical overlap between the different IDs is reflected on molecular level by the identification of similar multilocus methylation defects in different phenotypes. In particular, in growth retarded patients, it is sometimes difficult to decide which ID-specific test should be applied.

### Multilocus imprinting disturbances, a common finding in IDs

The correlation between aberrations at specific imprinted genes and distinct congenital disorders was generally accepted for nearly 20 years, but there are growing numbers of reports on patients with generally disturbed imprinting patterns (MLID) (Table 1) (for review: [26]). These patients often exhibit a specific ID phenotype, for example, BWS, but molecular testing reveals that aberrant methylation does not affect only the disease-specific imprinted loci (for example, 11p15 in BWS) but also other imprinted regions. Remarkably, patients with opposite phenotypes like BWS (overgrowth) and SRS (growth retardation) can share some aberrant methylation patterns in lymphocytes (lymphocyte DNA is the most frequently and often the only analysed tissue), and to add a layer of complexity, epigenotype anomalies can vary for the same individual depending on the studied tissue. This has been so far investigated in SRS patients with 11p15 ICR1 LOM identified initially in leukocytes. Another example is the phenotype of an upd(7q)mat carrier with hypomethylation in 14q32 who was initially diagnosed as SRS (typical for upd(7q)mat but then exhibited a phenotype suggestive for TS14 [27].

In summary, the clinical picture in patients with methylation aberrations affecting more than one imprinted locus can be different from that of patients with single epimutations, but is not necessarily so. As a result, both patients with typical ID phenotypes as well as those with unusual clinical features should be tested for MLID.

Two genetic mechanisms causing aberrant methylation at imprinted loci have been identified: *cis*- and *trans*-acting alterations. *Cis-acting alterations* affect DNA sequences which are physically localised adjacent to a structural gene or its control regions and interact with them. Examples for *cis*-acting mutations are rare deletions of CTCF-binding sites and mutations affecting the OCT- and SOX-binding elements in 11p15 which mediate the regulation of ICR1: such mutations affect up to 20% of BWS patients with 11p15 ICR1 GOM [28], but the BWS phenotype is expressed only if the mutation affects the maternal chromosome 11 [29,30]. *Trans-acting factors* also influence the expression of genes, but they act by intermediary diffusible molecules (proteins, RNAs). Their genes can be localised on the same chromosome as the target gene or elsewhere in the genome. Several trans-acting factors have been postulated to cause MLID (for review: [26]), and indeed, DNA mutations in factors involved in the imprinting cycle have been reported [31-33].

In addition to these monogenic causes of aberrant imprinting, the identification of patients with MLID corroborates the hypothesis of an ‘imprinted gene network’, that is, a network of interacting imprinted genes and regions [34]. By this network, the disturbance of one member alters the regular expression of the others.

### The COST Action BM1208: EUCID.net - a network of European groups working in the field of IDs

Despite their common underlying (epi)genetic aetiologies, IDs are usually studied separately by small groups working in isolation, and the basic pathogenesis and long-term clinical consequences of IDs remain largely unknown. Efforts to elucidate the aetiology of IDs are currently fragmented across Europe, and standardisation of diagnostic and clinical management is lacking.

To overcome this fragmentation and to achieve a consensus in diagnostic and treatment of IDs, European groups working on IDs and epigenetic regulation have established a network, called EUCID.net (European network for human congenital imprinting disorders; www.imprinting-disorders.eu), which, for the first time, draws together researchers of all eight known human IDs in an interdisciplinary activity, working to advance understanding of the pathophysiology with the major aim of translating this knowledge to improvement of diagnostic and clinical management for the benefit of the patients and their families. The Action will harmonise a common system for clinical and molecular classification as well as nomenclature of IDs, develop guidelines for treatment through consensus, create standard operating procedures (SOPs) for diagnosis based on best current practice, coordinate databases held in different countries to make them compatible and useful as a springboard for collective research initiatives, identify new imprinting disorders through collaborative effort, educate researchers and stimulate translational exchange. These networking activities have become possible with funding of COST, the European Cooperation in Science and Technology, (COST Action BM1208).

### Objectives of EUCID.net

The objectives of the network will be realised in five working groups (WGs) described below. The activities will run in three interdependent directions: (a) Clinical experts are undertaking the challenging task of standardisation and harmonisation of clinical phenotyping and medical management of IDs, providing guidelines for IDs’ clinical assessment and management across Europe. (b) Experts in molecular diagnosis are undertaking the standardisation of molecular diagnosis of IDs and development of consistent reproducible molecular testing. (c) A coordinated European infrastructure of data sharing (clinical and molecular data) and samples for genetic and epigenetic study of IDs will be created (with the possibility to link clinical, genetic and epigenetic data). This will be an important step towards improving the standard of care for IDs in Europe and uncovering the genetic/epigenetic bases of the disorders.

### WG1 - European clinical integration

By European wide cooperation and coordination, this WG will gather Europe-wide experience in the clinical and metabolic characteristics and management practices of ID patients to provide a comprehensive clinical review of IDs. This will enable the development of standardised recording of phenotypes across centres and the development of a disease classification enabling future improvements in diagnosis. We will also strive for a current consensus on clinical management guidelines on which to build as new developments emerge. With the publication of disease-specific clinical utility cards, the first step has been undertaken [35-40].

### WG2 - molecular biology

This multidisciplinary WG will unify recording of samples to create a virtual biobank for IDs across Europe, creating pan-European resources for the study of the (epi)genetic basis of IDs. The heterogeneity and complexity of IDs demands the availability of large patient cohorts which has not been available until now. Furthermore, this WG will attempt to unify the types of the biological material obtained from affected families (DNA, RNA, transformation of lymphocytes). This will allow the identification of factors commonly involved in the aetiology of IDs. This WG will focus on activities aiming on the identification of new genes, *cis*-acting control elements and epigenetic *trans*-acting factors associated with and/or causing IDs as a prerequisite for an understanding of pathophysiological mechanisms. Unravelling the cross talk between known and new ID genes can place these disorders in an (epi)genomic perspective. This WG will coordinate genomic, epigenomic, proteomic and transcriptomic studies by high-throughput assays. By data exchange, these new findings and techniques will be implemented in clinical and diagnostic application.

### WG3 - molecular diagnostics

WG3 will discuss important technical aspects related to molecular diagnosis of IDs, aiming to provide a harmonised testing algorithm for IDs. The partners in WG3 will implement innovative diagnostic algorithms by improvement of existing ID-specific tests and by development of new tests for specific IDs. The diagnostic utility of these techniques will be validated, and standardised algorithms will be progressively incorporated into diagnostic regimes through collective experience. Reference panels and quality control measures will be established in cooperation with the European Molecular Quality Network (EMQN). It is expected that most European experts in the field of IDs are going to participate in this effort and they will collaborate with experts around the world, thus turning this COST Action into a global initiative.

### WG4 - capacity building

Being the springboard for new research ideas, this WG will use the combined experiences of COST members to initiate new directions and projects in ID research responding to research calls through the preparation and submission of grant proposals to European and international funding agencies. Furthermore, networking activities and short-term scientific missions will be organised to strengthen the interdisciplinary and transnational activities.

### WG5 - dissemination

This WG will design and coordinate outreach meetings with patient groups and dissemination activities. It will receive input from the other WGs and channel their scientific efforts into public dissemination. The publication of new disease classification systems and clinical guidelines, together with coordinated diagnostics, will represent a major objective for delivering individualized therapeutic management. This WG will promulgate the results, guidelines and clinical classifications to physicians, scientists and patients’ organisations. WG5 will have close links with national patients’ organisations.

### Organisation

At the beginning in May 2013, 25 groups from 11 European countries have been part of EUCID.net, including academic groups, SMEs and patients’ organisations. Until July 2014, additional groups have joined the network, and it currently includes 45 groups from 22 European countries. Furthermore, there are close links and exchanges with experts from Australia, Canada, Japan, South America and the USA.

**Figure 2:**
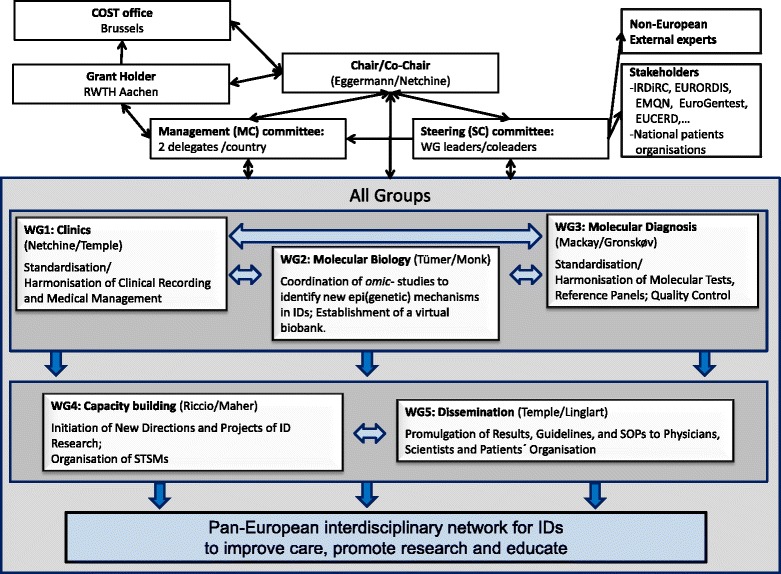
**Structure and activities of the EUCID.net/COST BM1208, all aiming on networking of researchers, clinicians, SMEs and patients organisation working in the field of IDs.**

Based on the COST rules, the EUCID.net includes the following partcipants (Figure 2): A Chair and a Vice-Chair, both elected, who preside over the *management committee (MC)*. The *MC* coordinates the key issues of the Action. It consists of two representatives per participating country, including the Coordinator and Co-Coordinator of each WG. It is responsible for the allocation of funds and oversees for the overall strategy of the network. The MC manages operations of the WGs, the programme of international symposia, as well as training and exchange programmes. The MC fosters the exchange of scientific knowledge and active collaborators. Due to its smaller size, the *steering committee (SC)*, consisting of the WG co/coordinators, represents a more flexible instrument that allows the close monitoring of the progress of the Action and acts as a link between the WGs and the different groups. Under the directions of the MC, the SC is furthermore responsible for the interaction with existing platforms in Europe and across the world and relevant stakeholders (for example, IRDiRC, EURORDIS, Orphanet, EMQN, EUCERD). Each of the five WGs is chaired by a WG leader (Coordinator) and a co-leader who have been elected by the MC during the kick-off meeting. Leader and co-leader are responsible for the coordination, organisation and supervision of the WG’s meetings. Each WG is constituted by different teams, but the teams can be involved in different WGs. All members of each WG will meet once a year to establish the guidelines and SOPs as well as to exchange scientific knowledge and data. The investigators of each WG communicate through regular conference calls and web-based interfaces. All five WGs are interrelated and interact through the SC. WG 1 is closely related to WG 2 and 3, establishing tools and guidelines for phenotype characterization. In turn, WG 2 and 3 are connected to deciphering the molecular basis of IDs and to translate the achieved knowledge into diagnostic application. Furthermore, they contribute to the ID classification and development of clinical guidelines in WG 1. All efforts from WG1 3 support and stimulate the activities in WG 4 to initiate new directions and projects. WG1 4 support WG 5 in the promulgation of the data and achieved knowledge, *inter alia* by implementation of WG specific information on the website in a password protected area.

High priority is given to *Early Stage Researchers (ESRs)* and *Short-Term Scientific Missions (STSMs)* for maximizing the exchange of experience among the participants as exchanging ideas and knowledge across borders will lead to more successful projects. The *ID training school* is a further central instrument to pursue these aims.

## Conclusions

Imprinting disorders are underdiagnosed, and currently available diagnostic and management protocols are suboptimal. Improvements in the diagnosis and management of rare diseases are greatly facilitated by international collaboration. EUCID.net aims to promote better clinical care and scientific investigation of imprinting disorders by establishing a concerted multidisciplinary alliance of clinicians, researchers, patients and families. By encompassing all IDs and establishing a wide ranging, open and collaborative network, EUCID will bring together a wide variety of expertise and interests to engender new collaborations and initiatives. It is very much hoped that epigeneticists with an interest in imprinting disorders will wish to participate in EUCID.net (contact: teggermann@ukaachen.de).
